# Retraction Note: LncRNA TUBA4B functions as a competitive endogenous RNA to inhibit gastric cancer progression by elevating PTEN via sponging miR-214 and miR-216a/b

**DOI:** 10.1186/s12935-022-02823-4

**Published:** 2022-12-14

**Authors:** Jianbo Guo, Yan Li, He Duan, Lu Yuan

**Affiliations:** Department of General Surgery, The Fourth Affiliated Hospital of China Medical University, 4 Chongshan East Street, Shenyang, 110032 Liaoning People’s Republic of China

**Retraction Note: Cancer Cell Int (2019) 19:156** https://doi.org/10.1186/s12935-019-0879-x

The Editors-in-Chief have retracted this article at the corresponding author's request. After publication, the authors found that the data could not be replicated. This was caused by mislabelling of patient blood samples and the use of contaminated cell lines (SGC-7901, BGC-823 and MGC-803) in this study.

The authors therefore no longer have confidence in the presented results.

All authors agree to this retraction.

